# Alkaline phosphatase decline and pain response as predictors of overall survival benefit in patients treated with radium-223: a post hoc analysis of the REASSURE study

**DOI:** 10.1038/s41416-024-02927-w

**Published:** 2025-01-09

**Authors:** Joe M. O’Sullivan, Daniel Heinrich, Elena Castro, Saby George, Sabina Dizdarevic, Sergio Baldari, Markus Essler, Igle Jan de Jong, Secondo Lastoria, Peter G. Hammerer, Bertrand Tombal, Nicholas D. James, Jeff Meltzer, Per Sandström, Oliver Sartor

**Affiliations:** 1 Patrick G. Johnston Centre for Cancer Research, Queen’s University Belfast and Northern Ireland Cancer Centre, Belfast, UK; 2 Department of Medical and Radiation Oncology, Innlandet Hospital Trust, Gjøvik, Norway; 3 Hospital Universitario 12 de Octubre, Madrid, Spain; 4 Roswell Park Cancer Institute, Buffalo, NY USA; 5 University Hospitals Sussex NHS Foundation Trust, Clinical Imaging Science Centre, Brighton & Sussex Medical School, University of Sussex and Brighton, Brighton, UK; 6 Nuclear Medicine Unit, Department of Biomedical and Dental Sciences and Morphofunctional Imaging, University of Messina, Messina, Italy; 7 Department of Nuclear Medicine, University Hospital Bonn, Bonn, Germany; 8 Department of Urology, University Medical Center Groningen, Groningen, The Netherlands; 9 IRCCS National Cancer Institute, Fondazione Senatore G. Pascale, Naples, Italy; 10 Department of Urology, Academic Hospital, Braunschweig, Germany; 11 Cliniques Universitaires Saint Luc, Brussels, Belgium; 12 The Institute of Cancer Research, London, UK; 13 Bayer HealthCare Pharmaceuticals, Whippany, NJ USA; 14 Bayer A/S, Copenhagen, Denmark; 15 Mayo Clinic, Rochester, MN USA

**Keywords:** Prostate cancer, Prostate cancer

## Abstract

**Background:**

Alkaline phosphatase (ALP) declines and pain responses can occur during radium-223 (^223^Ra) treatment, but their association with treatment outcomes is unclear.

**Methods:**

For patients with metastatic castration-resistant prostate cancer treated with ^223^Ra in the REASSURE study, we investigated whether ALP decline (Week 12) and/or pain response (during treatment) are associated with improved overall survival (OS). The Brief Pain Inventory-Short Form (BPI-SF) was used to assess pain at baseline and pain response (in patients with baseline BPI-SF score ≥2).

**Results:**

Of 785 patients with baseline and Week 12 ALP measurements, 779 were eligible for the OS analyses. Overall, 80% of patients had an ALP decline. Median OS was longer in patients with than without an ALP decline (18.1 versus 14.2 months; HR 0.74; 95% CI 0.60–0.92). In patients with an ALP decline, there was no clear OS difference between those with versus without a pain response. For patients without ALP decline, median OS was longer in those with versus without a pain response (16.2 versus 10.9 months; HR 0.50; 95% CI 0.32–0.77).

**Conclusions:**

Decreases in ALP and/or pain during ^223^Ra treatment are associated with improved OS. This may help support clinical decisions.

**Clinical trial registration:**

ClinicalTrials.gov identifier NCT02141438.

**Figure:**
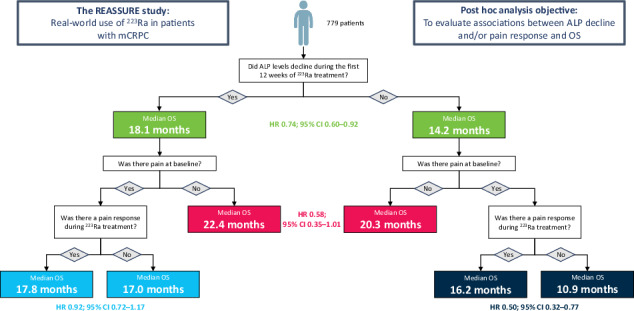
Analyses from the radium-223 REASSURE global study suggest that declines in alkaline phosphatase and pain during treatment may predict longer survival in patients with advanced prostate cancer and may help doctors make decisions with their patients.

## Background

Bone metastases occur in the majority of patients with metastatic castration-resistant prostate cancer (mCRPC) [1] and can lead to skeletal complications and pain, with a negative impact on patient quality of life (QoL) and survival [2]. Radium-223 dichloride (^223^Ra) is an alpha particle-emitting radiotherapeutic approved for the treatment of mCRPC with symptomatic bone metastases and no known visceral metastases [3, 4]. This approval was based on the findings of the international phase 3, randomised, double-blind ALSYMPCA study in patients with mCRPC, with or without prior chemotherapy, and with symptomatic bone metastases. In this study, ^223^Ra improved overall survival (OS) versus placebo when used in combination with best standard of care (14.9 versus 11.3 months; hazard ratio [HR] 0.70; 95% confidence interval [CI] 0.58–0.83, P < 0.001) [5], and was well tolerated [6]. ^223^Ra treatment also delayed time to skeletal-related events and improved health-related QoL [5, 7, 8].

Assessing treatment response to determine whether therapy should be continued in patients with mCRPC requires consideration of factors including prostate-specific antigen (PSA) levels, radiographic progression, bone scans, and clinical deterioration [9]. Despite prolonged survival, PSA responses are uncommon with ^223^Ra [5], highlighting the need to identify alternative markers for determining treatment outcomes. Several prognostic markers have been assessed in patients with mCRPC receiving treatment with ^223^Ra, including imaging techniques such as the bone scan index, although none have been validated to date [10].

During the development of bone metastases, prostate cancer cells and cancer-associated osteoblasts secrete elevated levels of alkaline phosphatase (ALP), with baseline levels of this protein being prognostic of OS in patients with mCRPC [11]. Similarly, pain is often associated with disease progression and is an indicator of OS in patients with mCRPC [12]. However, whether ALP or pain are markers of OS, specifically in patients with mCRPC treated with ^223^Ra, is less well established.

The REASSURE study (NCT02141438) is an ongoing, prospective, non-interventional study assessing the long-term safety of ^223^Ra in patients with mCRPC in routine clinical practice [13]. In this post hoc analysis of the second interim analysis of REASSURE, we investigated the associations of ALP decline and/or pain response during ^223^Ra treatment with OS to explore their potential as markers of therapeutic efficacy.

## Methods

### Study design and patients

A post hoc analysis was conducted using data collected at the preplanned second interim analysis of the REASSURE study. REASSURE is a global observational study being conducted at 150 sites worldwide. Full methodological details have been reported previously [13]. In brief, eligible patients had mCRPC with bone metastases and were scheduled for treatment with ^223^Ra by their physician. Exclusion criteria included prior treatment with ^223^Ra for any reason, current treatment in clinical trials, including other ^223^Ra studies, and planned concomitant use of other systemic radiopharmaceuticals for any reason.

### Procedures

The decision to treat with ^223^Ra was made independent from, and prior to, the provision of study information. ^223^Ra treatment was prescribed and administered as part of routine clinical practice and according to local health authority-approved labels. Patients with ALP measurements at both baseline and Week 12 (closest value between Weeks 8 and 16) and who had received at least one dose of ^223^Ra were included in this analysis. Patients were grouped on the basis of whether they had no ALP decline or any ALP decline from baseline at Week 12. Pain measurements were documented at baseline and during ^223^Ra treatment using the worst pain in the last 24 hours item of the Brief Pain Inventory-Short Form (BPI-SF) questionnaire. Patients were grouped into no pain at baseline (baseline BPI-SF score of 0–1) or pain at baseline (baseline BPI-SF score ≥2). Pain responses were categorised into two groups: pain response (a decrease of ≥2 points in BPI-SF score) and no pain response (no decrease or decrease of <2 points in BPI-SF score) during treatment.

### Outcomes

As reported previously, the primary endpoints of the study were to describe the short- and long-term safety profile of ^223^Ra [13]. Secondary endpoints were OS, worst pain score, and pain interference score over time. This analysis describes associations of ALP decline and pain response with OS during ^223^Ra treatment.

### Statistical analysis

Descriptive summaries were provided for mean, standard deviation, median, minimum and maximum for continuous variables and counts and percentages for categorical variables. In addition, descriptive statistics for Kaplan-Meier estimates were provided with 95% CIs. OS was calculated from the start of ^223^Ra treatment for eligible patients at the designated landmark timepoint of 12 weeks. Median OS and corresponding HRs and 95% CIs were reported for subgroups of ALP decline with pain status/response using a Cox regression analysis.

**Fig. 1:**
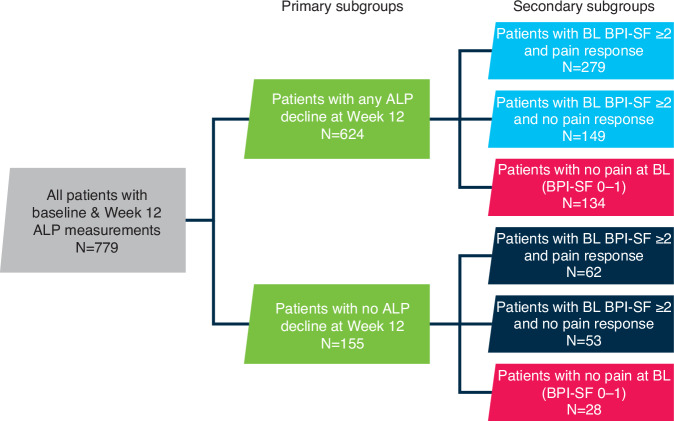
Patient subgroups analysed. Of 785 patients with baseline and Week 12 ALP measurements, 779 were eligible for inclusion at the designated landmark timepoint of 12 weeks and were included in the analysis. ALP alkaline phosphatase, BL baseline, BPI-SP brief pain inventory short form.

Post hoc comparisons of OS were made for the primary patient subgroups of any ALP decline versus no ALP decline from baseline at Week 12, and for the following six secondary patient subgroups: any ALP decline versus no ALP decline in patients with no pain at baseline (BPI-SF 0–1); pain response versus no pain response in patients with baseline pain (BPI-SF ≥ 2) and any ALP decline; and pain response versus no pain response in patients with baseline pain (BPI-SF ≥ 2) and no ALP decline (Fig. 1).

In a separate post hoc analysis, patients treated with ^223^Ra were grouped by baseline ALP ≤ 147 U/L versus >147 U/L and then by any ALP decline versus no decline at Week 12. The 147 U/L cut-off was selected based on the highest reported upper limit of normal for ALP [14]. The association of ALP decline with OS was assessed in the ≤147 U/L and >147 U/L groups separately. HRs from Cox regression models, including selected baseline variables, were provided with 95% CIs. A stepwise approach for model selection was used. Baseline covariates at the 0.10 two-sided level included in the model were: ALP categories; age; log PSA, haemoglobin; and prior therapies ( ≥ 1 versus 0). Eastern Cooperative Oncology Group Performance Status (ECOG PS) (0–1, ≥2) was included, but was not selected at the 0.10 two-sided level.

## Results

In total, 1465 patients were enroled from August 2014 until the data cut-off of March 2019, of whom 785 (54%) had ALP measurements at both baseline and Week 12 (closest value between Weeks 8 and 16). Baseline demographics and clinical characteristics are shown in Table 1. There were few relevant differences observed among the primary subgroups (i.e., ‘No ALP decline’ and ‘Any ALP decline’). Among these, ALP levels >147 U/L were seen in a greater proportion of patients in the ‘Any ALP decline’ group versus the ‘No ALP decline’ group (47% versus 27%). The proportion of patients who completed six ^223^Ra injections was greater in the ‘Any ALP decline’ group than the ‘No ALP decline’ group (74% versus 59%). However, these two groups had broadly similar proportions of patients with self-reported BPI-SF scores of >1 for worst pain in last 24 hours (69% versus 74%).

**Table 1:** Baseline characteristics and ^223^Ra treatment compliance of evaluable patients (N = 785)

	Primary subgroups		Secondary subgroups					
			No BL pain (BPI-SF 0–1)		BL pain (BPI-SF ≥ 2)			
					Any ALP decline		No ALP decline	
	Any ALP decline	No ALP decline	Any ALP decline	No ALP decline	Pain response	No pain response	No pain response	Pain response
Characteristic, (%)	(N = 628)	(N = 157)	(N = 135)	(N = 29)	(N = 280)	(N = 150)	(N = 54)	(N = 62)
**Baseline characteristics**								
Age group (years)								
≥65	537 (86)	135 (86)	125 (93)	26 (90)	231 (83)	128 (85)	48 (89)	51 (82)
ECOG PS^a^								
0–1	516 (82)	131 (83)	113 (84)	22 (76)	226 (81)	127 (85)	42 (78)	56 (90)
≥2	80 (13)	18 (12)	10 (7)	2 (7)	39 (14)	22 (15)	10 (19)	5 (8)
Hgb^a^								
<10 g/dL	39 (6)	9 (6)	8 (6)	3 (10)	12 (4)	14 (9)	3 (6)	3 (5)
≥10 g/dL	582 (93)	147 (94)	127 (94)	26 (90)	265 (95)	133 (89)	51 (94)	58 (94)
PSA^a^								
0–100 ng/mL	358 (57)	79 (50)	81 (60)	13 (45)	163 (58)	81 (54)	26 (48)	33 (53)
>100 ng/mL	198 (32)	68 (43)	43 (32)	13 (45)	84 (30)	47 (31)	27 (50)	23 (37)
ALP								
≤147 U/L	331 (53)	115 (73)	89 (66)	24 (83)	133 (48)	78 (52)	38 (70)	45 (73)
>147 U/L	297 (47)	42 (27)	46 (34)	5 (17)	147 (53)	72 (48)	16 (30)	17 (27)
Worst pain in last 24 hours (BPI-SF score)^a^								
0–1	135 (22)	29 (19)	135 (100)	29 (100)	0 (0)	0 (0)	0 (0)	0 (0)
>1	430 (69)	116 (74)	0 (0)	0 (0)	280 (100)	150 (100)	54 (100)	62 (100)
Number of prior life-prolonging therapies^b^ for mCRPC								
0	250 (40)	53 (34)	55 (41)	8 (28)	117 (42)	55 (37)	17 (32)	22 (36)
≥1	378 (60)	104 (66)	80 (59)	21 (72)	163 (58)	95 (63)	37 (69)	40 (65)
Number of prior completed chemotherapies for mCRPC								
0	396 (63)	93 (59)	95 (70)	17 (59)	177 (63)	88 (59)	27 (50)	42 (68)
≥1	232 (37)	64 (41)	40 (30)	12 (41)	103 (37)	62 (41)	27 (50)	20 (32)
^**223**^**Ra treatment compliance**								
Number of ^223^Ra injections								
<6	161 (26)	65 (41)	23 (17)	13 (45)	68 (24)	57 (38)	30 (56)	17 (27)
6	467 (74)	92 (59)	112 (83)	16 (55)	212 (76)	93 (62)	24 (44)	45 (73)

Values may not add up to 100% due to rounding. ^a^Values do not add up to 100%; the remaining patients had missing data. ^b^Docetaxel, cabazitaxel, abiraterone acetate, enzalutamide, sipuleucel-T.

*ALP* alkaline phosphatase, *BL* baseline, *BPI-SF* Brief Pain Inventory-Short Form, *ECOG PS* Eastern Cooperative Oncology Group Performance Status, *Hgb* haemoglobin, *mCRPC* metastatic castration-resistant prostate cancer, *PSA* prostate-specific antigen, ^223^*Ra* radium-223.

**Fig. 2:**
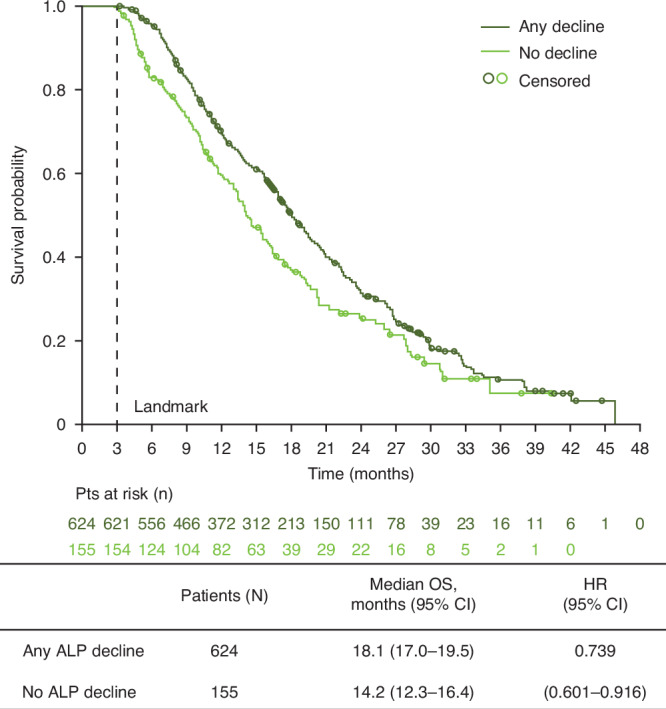
Overall survival in patients with or without ALP decline at Week 12. ALP alkaline phosphatase, CI confidence interval, OS overall survival, HR hazard ratio.

Of 785 patients with baseline ALP measurements, 779 were eligible for inclusion at the designated landmark timepoint of 12 weeks and were included in the analysis (one patient had died and five patients could not be included and were censored). The number of patients analysed in each subgroup is shown in Fig. 1. In the two primary subgroups, 624 patients (80%) had a decline in ALP at Week 12 and 155 patients (20%) had no decline. Patients with ALP decline had longer median OS than patients with no ALP decline (18.1 versus 14.2 months; HR 0.74; 95% CI 0.60–0.92) (Fig. 2).

**Fig. 3:**
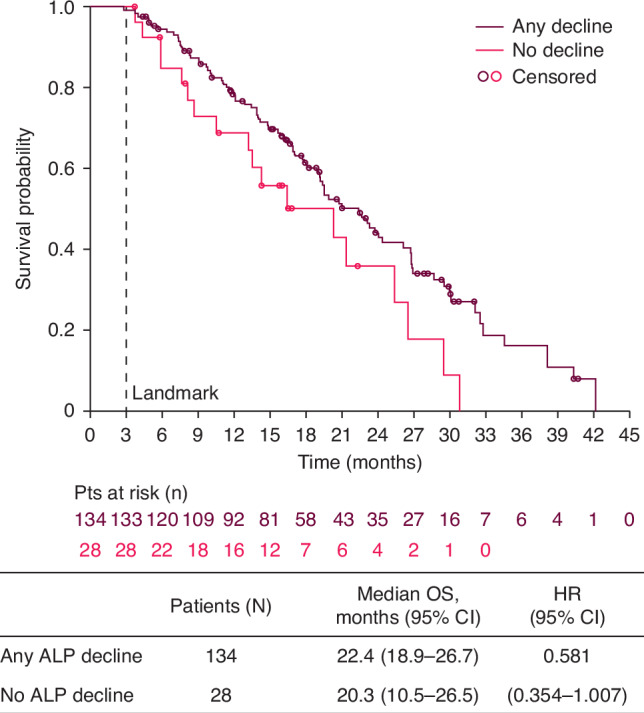
Overall survival in patients with no pain at baseline (BPI-SF score 0–1), with or without ALP decline at Week 12. ALP alkaline phosphatase, BPI-SF Brief Pain Inventory-Short Form, CI confidence interval, OS overall survival, HR hazard ratio.

**Fig. 4:**
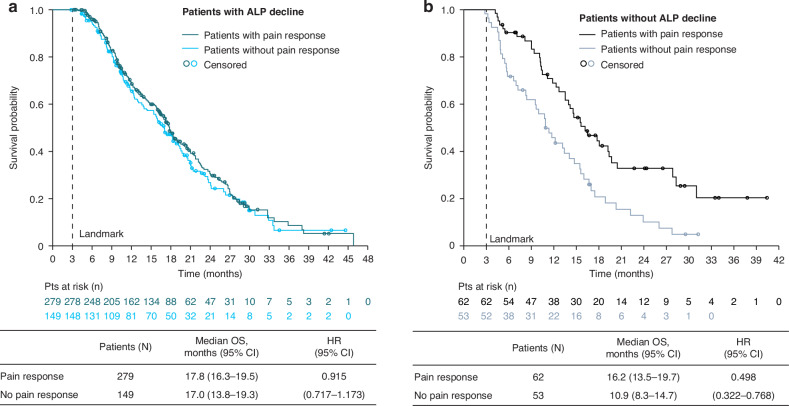
Overall survival in patients with pain at baseline (BPI-SF ≥ 2), with (**a**) or without (**b**) ALP decline at Week 12, grouped according to pain response. ALP alkaline phosphatase, BPI-SF, Brief Pain Inventory-Short Form; CI confidence interval, OS, overall survival, HR hazard ratio.

For secondary subgroups, among patients with no pain at baseline (BPI-SF score 0–1), median OS trended longer in patients who had any ALP decline compared with those with no ALP decline (22.4 versus 20.3 months; HR 0.58; 95% CI 0.35–1.01) (Fig. 3). In patients with pain at baseline (BPI-SF score ≥2) who had any ALP decline, median OS was similar regardless of pain response (17.8 months in those with a pain response versus 17.0 months in those without; HR 0.92; 95% CI 0.72–1.17) (Fig. 4a). In contrast, in patients with pain at baseline (BPI-SF score ≥2) who had no ALP decline, median OS was longer for those who had a pain response versus no pain response (16.2 versus 10.9 months; HR 0.50; 95% CI 0.32–0.77) (Fig. 4b).

**Fig. 5:**
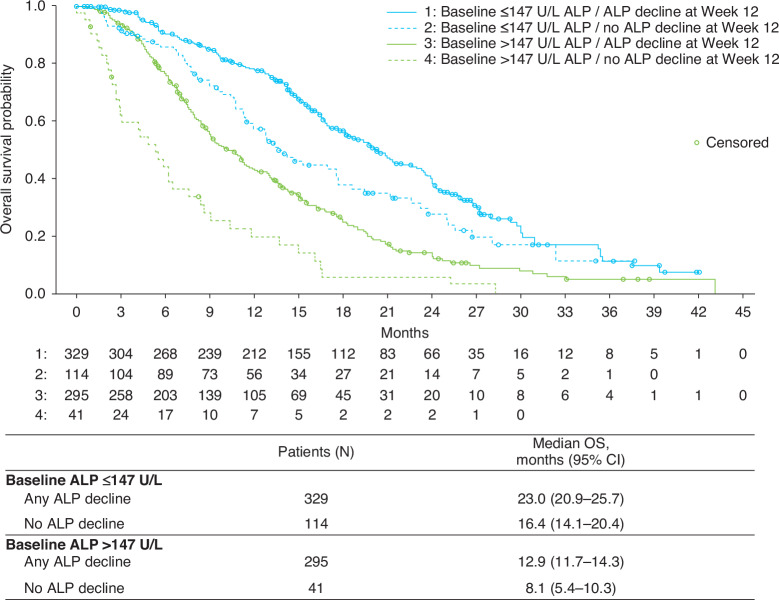
Median overall survival in patients with or without ALP decline at Week 12 according to baseline ALP level. ALP alkaline phosphatase, CI confidence interval, OS overall survival.

Associations between ALP decline and median OS were also evaluated in patients grouped by baseline ALP level ( ≤ 147 U/L and >147 U/L). Baseline and clinical characteristics for these subgroups are reported in Supplementary Table 1. Median OS was notably longer in patients whose baseline ALP was ≤147 U/L versus >147 U/L, regardless of whether or not they had ALP decline at Week 12 (Fig. 5). However, within each group, patients who had an ALP decline had longer OS compared with those without ALP decline (Fig. 5, Supplementary Table 2). In patients with baseline ALP ≤ 147 U/L, ALP decline was associated with increased OS after adjustment for the baseline variables of age, PSA, haemoglobin and prior life-prolonging therapies. In patients with baseline ALP > 147 U/L, there were two-way interaction effects of ALP decline with age, PSA and haemoglobin that were associated with increased OS after adjustment for prior life-prolonging therapies (Supplementary Table 2). Some covariates were associated with decreased OS, including having ≥1 prior life-prolonging therapy (in both the ≤147 U/L and >147 U/L groups) and increasing age and PSA (in the ≤147 U/L group) (Supplementary Table 2).

## Discussion

Monitoring response to prostate cancer treatment is important for both physicians and patients, with PSA response typically used [15, 16]. While PSA declines are common following treatment with androgen receptor pathway inhibitors and taxanes [17–20], this is not the case for ^223^Ra or sipuleucel-T [21]. Although there are currently no established response markers for these last two agents, potential markers of response have been extensively investigated [22–25]. For ^223^Ra, these potential markers include ALP and pain [22–24, 26–31], although no studies to date have looked at these two markers in combination. Here we investigated the role of ALP and pain in predicting survival in patients treated with ^223^Ra in routine clinical practice.

In our analysis, ALP decline at Week 12 was associated with improved OS. In patients with an ALP decline and pain at baseline, there was no clear OS difference between those with versus those without a pain response. However, in patients who did not experience a decline in ALP and who had pain at baseline, pain response during treatment was associated with longer OS. These results indicate that both ALP decline and pain response may be potential markers for assessing clinical benefit in patients receiving ^223^Ra therapy. The best marker combination for longer OS was no pain at baseline and ALP decline during treatment. Survival was shortest in patients who had pain at baseline but had no ALP decline at Week 12 and no pain response during treatment, suggesting it may be advisable to perform further clinical evaluation in such patients.

Consistent with our findings, several retrospective studies (N > 50) in patients treated with ^223^Ra have shown that any ALP decline [22–24] or an ALP reduction to normal range [27] is prognostic for OS, and that elevated baseline ALP (i.e., >150 U/L [32], ≥115 U/L [23, 33], ≥220 U/L [24] and >130 U/L [22]) is associated with worse OS. An additional study indicated that an elevated baseline ALP ( > 115 U/L) without a ≥ 10% decline in ALP after the first ^223^Ra dose is also prognostic of worse OS [33]. Furthermore, a post hoc analysis of data from ALSYMPCA found declines from baseline in total ALP correlated with longer OS, although did not meet statistical requirements of a surrogate marker [21].

The association of OS with either baseline pain or pain response in patients treated with ^223^Ra has been evaluated in several real-world studies [27–31]. Although some of these are limited by small patient numbers (N < 50) [28–30], the largest studies (N = 110 [31] and 160 [27]) found that pain response during ^223^Ra treatment was associated with OS (in univariate but not multivariate analyses) [31] and pain relief was associated with improved OS [27]. Results from these studies and our current study suggest that, like ALP, pain could also potentially be used to determine the likely survival benefit patients may experience with ^223^Ra treatment. Some studies in patients with mCRPC have shown that absence/lower levels of pain are associated with longer OS [34–36], a finding that has not been seen for some other cancers [37]. Moreover, another study found higher pain, particularly bone pain, to be associated with higher mortality in patients with advanced prostate cancer, with this association being stronger in castrate-resistant disease than metastatic hormone-sensitive disease [38]. Notably, as studies have indicated that pain is detrimental to QoL [39, 40] and changes in QoL can be prognostic of survival [41], it is possible that pain control may improve QoL and thus survival.

Consistent with other real-world analyses [32, 42], our analyses of patients with normal or high baseline ALP levels found certain covariates (having received prior life-prolonging therapy, increasing age and increasing PSA) to be associated with decreased OS. Analyses to assess these and other covariates in patients stratified according to both pain and ALP decline may be of interest (i.e., perhaps patients with pain at baseline and no ALP decline or pain response may have received more prior therapies).

The findings of this analysis should be considered in the context of its limitations. These include the fact that this is a post hoc analysis of an observational, non-interventional study without a control group. However, the study is strengthened by the relatively large cohort of patients treated with ^223^Ra in routine clinical practice.

Our findings indicate that ALP decline and pain response may be considered as potential predictive markers of survival after treatment with ^223^Ra, and may therefore help support clinical decision making.

## Supplementary information

Supplementary information

## Data Availability

Availability of the data underlying this publication will be determined according to Bayer’s commitment to the EFPIA/PhRMA “Principles for responsible clinical trial data sharing”. This pertains to scope, timepoint and process of data access. As such, Bayer commits to sharing upon request from qualified scientific and medical researchers patient-level clinical trial data, study-level clinical trial data, and protocols from clinical trials in patients for medicines and indications approved in the United States (US) and European Union (EU) as necessary for conducting legitimate research. This applies to data on new medicines and indications that have been approved by the US and EU regulatory agencies on or after January 01, 2014. Interested researchers can use www.clinicalstudydatarequest.com to request access to anonymised patient-level data and supporting documents from clinical studies to conduct further research that can help advance medical science or improve patient care. Information on the Bayer criteria for listing studies and other relevant information is provided in the Study sponsors section of the portal. Data access will be granted to anonymised patient-level data, protocols and clinical study reports after approval by an independent scientific review panel. Bayer is not involved in the decisions made by the independent review panel. Bayer will take all necessary measures to ensure that patient privacy is safeguarded.
