# Supplementary material for: Alkaline phosphatase decline and pain response as predictors of overall survival benefit in patients treated with radium-223: a post hoc analysis of the REASSURE study
Source: Br J Cancer. 2025 Jan 9;132(4):354–60. doi: 10.1038/s41416-024-02927-w (PMC11833053; doi:10.1038/s41416-024-02927-w)
Supplement: Supplementary file 1 — Supplementary information [file 41416_2024_2927_MOESM1_ESM.docx]

**Supplementary appendix for “Alkaline phosphatase decline and pain response as predictors of overall survival benefit in patients treated with radium-223: a *post hoc* analysis of the REASSURE study”**

**Table of contents**

| Supplementary Table 1. Summary of baseline and clinical characteristics by baseline ALP levels and ALP decline | Page 2 | |
| --- | --- | --- |
| Supplementary Table 2. Comparisons of unadjusted and adjusted hazard ratios for overall survival | Page 3 | |
| Supplementary Table 3. List of ethics committees/institutional review boards that approved the study | Page 5 |  |
| Plain language summary | Page 11 | |

**Supplementary Table 1. Summary of baseline and clinical characteristics by baseline ALP levels and ALP decline (N=785)^a^**

|  | **Baseline ALP ≤147 U/L** | | **Baseline ALP >147 U/L** | |
| --- | --- | --- | --- | --- |
|  | **Any ALP decline** | **No ALP decline** | **Any ALP decline** | **No ALP decline** |
| **Characteristic, (%)** | **(N = 331)** | **(N = 115)** | **(N = 297)** | **(N = 42)** |
| Age group |  |  |  |  |
| <65 | 51 (15) | 15 (13) | 40 (13) | 7 (17) |
| ≥65 | 280 (85) | 100 (87) | 257 (87) | 35 (83) |
| ECOG PS^b^ |  |  |  |  |
| 0–1 | 291 (88) | 100 (87) | 225 (76) | 31 (74) |
| ≥2 | 26 (8) | 9 (8) | 54 (18) | 9 (21) |
| Hgb^b^ |  |  |  |  |
| <10 g/dL | 8 (2) | 4 (3) | 31 (10) | 5 (12) |
| ≥10 g/dL | 318 (96) | 110 (96) | 264 (89) | 37 (88) |
| PSA^b^ |  |  |  |  |
| 0–100 ng/mL | 68 (21) | 43 (37) | 130 (44) | 25 (60) |
| >100 ng/mL | 225 (68) | 62 (54) | 133 (45) | 17 (40) |
| Time from CRPC diagnosis to study entry | | | | |
| <1 year | 94 (28) | 22 (19) | 65 (22) | 14 (33) |
| ≥1 year | 146 (44) | 59 (51) | 148 (50) | 19 (45) |
| Missing | 91 (27) | 34 (30) | 84 (28) | 9 (21) |
| Number of prior life-prolonging therapies^c^ for mCRPC | | | | |
| 0 | 142 (43) | 38 (33) | 108 (36) | 15 (36) |
| ≥1 | 189 (57) | 77 (67) | 189 (64) | 27 (64) |
| Number of prior completed chemotherapies for mCRPC | | | | |
| 0 | 220 (66) | 71 (62) | 176 (59) | 22 (52) |
| ≥1 | 111 (34) | 44 (38) | 121 (41) | 20 (48) |

^a^785 of 1465 pts had baseline ALP measurements, of whom 779 pts had Week 12 ALP measurements: 443 patients had ALP levels ≤147 U/L and 336 had ALP levels >147 U/L at baseline.

^b^Values do not add up to 100%; the remaining patients had missing data.

^c^Prior life prolong therapies include abiraterone acetate, enzalutamide, docetaxel, cabazitaxel or sipuleucel-T.

ALP, alkaline phosphatase; CRPC, castration-resistant prostate cancer; ECOG PS, Eastern Cooperative Oncology Group Performance Status; Hgb, haemoglobin; mCRPC, metastatic CRPC; PSA, prostate-specific antigen.

**Supplementary Table 2. Unadjusted and adjusted hazard ratios for the association of ALP decline with overall survival**

| **Covariates** | **Adjusted HR**^a^ **(95% CI)** | **Unadjusted HR (95% CI)** |
| --- | --- | --- |
| **Patients with baseline ALP ≤147 U/L** | **N = 395** | **N = 443** |
| Any ALP decline vs no ALP decline | 0.672 (0.507–0.901) | 0.647 (0.494–0.855) |
| Age (5-year increase) | 1.152 (1.059–1.254) |  |
| Log PSA (10-fold increase, ng/mL) | 1.126 (1.043–1.218) |  |
| Prior life-prolonging therapies (≥1 vs 0) | 1.660 (1.237–2.248) |  |
| **Patients with baseline ALP >147 U/L** | **N = 300** | **N = 336** |
| Any ALP decline vs no ALP decline |  | 0.462 (0.331–0.663) |
| ALP decline interaction with PSA^b^  Age 73, log PSA 3.5, Hgb 11.9 | 0.218 (0.133–0.379) |  |
| Age 73, log PSA 4.7, Hgb 11.9 | 0.342 (0.241–0.498) |  |
| Age 73, log PSA 5.8, Hgb 11.9 | 0.512 (0.323–0.858) |  |
| ALP interaction with age^c^  Age 68, log PSA 4.6, Hgb 11.9 | 0.456 (0.296–0.737) |  |
| Age 73, log PSA 4.6, Hgb 11.9 | 0.331 (0.233–0.483) |  |
| Age 79, log PSA 4.6, Hgb 11.9  ALP interaction with Hgb^d^ | 0.225 (0.138–0.388) |  |
| Age 73, log PSA 4.6, Hgb 10.9 | 0.244 (0.161–0.382) |  |
| Age 73, log PSA 4.6, Hgb 11.9 | 0.320 (0.225–0.468) |  |
| Age 73, log PSA 4.6, Hgb 13.2 | 0.456 (0.288–0.758) |  |
| Prior life-prolonging therapies^e^ (≥1 vs 0) | 1.630 (1.223–2.194) |  |

^a^Baseline covariates included in the model were: ALP categories; age; log PSA, Hgb; and prior therapies (≥1 versus 0).

^b^HR is estimated for the interaction of ALP decline with log PSA. The estimates provided are at quartiles for log PSA at median age and median Hgb.

^c^HR is estimated for the interaction of ALP decline with age. The estimates provided are at quartiles for age at median log PSA and median Hgb.

^d^HR is estimated for the interaction of ALP decline with Hgb. The estimates provided are at quartiles for Hgb at median age and median log PSA.

^e^Life-prolonging therapies included abiraterone acetate plus prednisone/prednisolone, enzalutamide, docetaxel, cabazitaxel, and sipuleucel-T.

Note 1: For ALP >147, selection of baseline covariates, prior life-prolonging therapies as well as two-way interactions of ALP decline with age, log PSA and Hgb, was from a Cox regression stepwise model at the 0.10 two-sided level. The HR displayed is for the interaction effect of ALP decline at quartiles of one factor while holding the other interaction factors constant at the respective medians. The table shows that in presence of interactions in the model that the estimate of the HR for ALP decline varies with respect to different levels of age, log PSA and Hgb. ALP decline was associated with increased OS for these interaction effects and adjustment for prior life-prolonging therapies.

Note 2: For ALP ≤147, selection of baseline covariates ALP decline, age, log PSA and life-prolonging therapies was from a Cox regression stepwise model at the 0.10 two-sided level. ALP decline was associated with increased OS after adjusting for these other factors.

Note 3: Laboratory covariates: PSA units are ng/mL and Hgb units are g/dL. Log PSA values of 3.5, 4.6, 4.7, and 5.8 correspond to 33.1, 99.5, 110.0, and 330.3, respectively, on the original ng/mL scale.

ALP, alkaline phosphatase; CI, confidence interval; Hgb, haemoglobin; HR, hazard ratio; OS, overall survival; PSA, prostate-specific antigen.

**Supplementary Table 3. List of ethics committees/institutional review boards that approved the study**

| **Study country** | **Site name** | **City** | **Name of ethics committee/institutional review board that approved the study** | **Date of initial approval** |
| --- | --- | --- | --- | --- |
| Argentina | CSMT | Ciudad Autonoma de Buenos Aires | Comite de Etica en Investigacion Clinica - CEIC | 05-May-16 |
|  | Consultorio Dr. Korbenfeld | Ciudad Autonoma de Buenos Aires | Comite de Etica en Investigacion Clinica - CEIC | 24-Nov-16 |
|  | Centro Medico Austral | Ciudad Autonoma de Buenos Aires | Comite de Etica en Investigacion Clinica - CEIC | 02-Feb-17 |
|  | Centro Medico Arenales | Ciudad Autonoma de Buenos Aires | Comite de Etica en Investigacion Clinica - CEIC | 22-Feb-17 |
| Austria | Kepler Universitätsklinikum | Linz | Ethikkommission Medizinische Universität Wien | 18-Nov-14 |
|  | Ordensklinikum Linz - Barmherzige Schwestern Linz | Linz | Ethikkommission Krankenhaus der Barmherzigen Schwestern Linz | 09-Mar-15 |
|  | LKH Steyr | Steyr | Ethikkommission Medizinische Universität Wien | 18-Nov-14 |
|  | AKH Wien Onkologie | Wien | Ethikkommission Medizinische Universität Wien | 18-Nov-14 |
|  | LKH Leoben | Leoben | Ethikkommission Medizinische Universität Wien | 18-Nov-14 |
|  | KH der Barmherzigen Brüder Wien | Wien | Ethikkommission Medizinische Universität Wien | 18-Nov-14 |
|  | AKH Wien Urologie | Wien | Ethikkommission Medizinische Universität Wien | 18-Nov-14 |
|  | LKH Salzburg | Salzburg | Ethikkommission Medizinische Universität Wien | 18-Nov-14 |
| Belgium | ZNA Middelheim | Antwerpen | Not required (non-interventional study) | N/A |
|  | AZ St Jan Brugge | Brugge | Not required (non-interventional study) | N/A |
|  | AZ Groeninge Campus VL | Kortrijk | Not required (non-interventional study) | N/A |
|  | AZ Turnhout | Turnhout | Not required (non-interventional study) | N/A |
|  | UZ Brussel | Brussels | Not required (non-interventional study) | N/A |
|  | Jessa Ziekenhuis Campus Virga Jesse | Hasselt | Not required (non-interventional study) | N/A |
|  | St Pierre Ottignies | Ottignies | Not required (non-interventional study) | N/A |
|  | UCL Saint Luc | Sint-Lambrechts-Woluwe | Comité d'Ethique Hospitalo-Facultaire (ECPSO) | 13-Apr-15 |
|  | CHU Sart Tilman | Luik | Not required (non-interventional study) | N/A |
|  | CMSE Namur | Namen | Not required (non-interventional study) | N/A |
|  | Jules Bordet | Brussels | Not required (non-interventional study) | N/A |
|  | Kliniek Sint Jan | Brussels | Not required (non-interventional study) | N/A |
| Canada | Southlake Regional Health Centre | Newmarket, Ontario | Southlake Regional Health Centre Research Ethics Board | 15-Mar-16 |
|  | British Columbia Cancer Agency Branch | Vancouver, BC | University of British Columbia - British Columbia Cancer Agency Research Ethics Board | 09-May-16 |
|  | Lakeridge Health Oshawa | Oshawa, Ontario | Lakeridge Health - Research Ethics Board | 04-Apr-16 |
|  | SAHCSI Cancer Centre for the Southern Interior | Kelowna, BC | University of British Columbia - British Columbia Cancer Agency Research Ethics Board | 09-May-16 |
| Colombia | Clinica Vida | Medellin | Non-interventional study – only required approval at the study site level | 21-Dec-16 |
|  | Clinica Las Americas | Medellin | Non-interventional study – only required approval at the study site level | 29-Nov-18 |
|  | Clinica San Vicente De Paul | Medellin | Non-interventional study – only required approval at the study site level | 23-Oct-18 |
| Czech Republic | Krajska zdravotni, a.s. - Masarykova nemocnice v Usti nad Labem, o.z. | Usti nad Labem | Etická komise Krajské zdravotní a.s., Masarykovi nemocnice,o.z. | 17-Dec-14 |
|  | Všeobecná fakultní nemocnice v Praze | Praha 2 | Etická komise Všeobecné fakultní nemocnice v Praze | 22-Jan-15 |
|  | Thomayerova nemocnice | Praha 4-Krč | Etická komise IKEM a FTN | 14-Jan-15 |
| Denmark | Rigshospitalet | Copenhagen | No ethics committee submission/approval required for non-interventional studies | N/A |
| France | CHU Toulouse | Toulouse cedex 9 | No ethical committee approval required for individual sites. General approval for the whole study was granted by the French local authority | N/A |
|  | Hôpital Cochin | PARIS |  | N/A |
|  | Centre Hospitalier Métropole Savoie | CHAMBERY |  | N/A |
|  | Centre hospitalier Henri Mondor | Creteil Cedex |  | N/A |
|  | Groupement Hospitalier Est | BRON CEDEX |  | N/A |
| Germany | Universitätsklinikum Jena | Jena | Ethikkommission der  Friedrich-Schiller-Universität Jena | 03-Jun-14 |
|  | Dietrich-Bonhoeffer-Klinikum Neubrandenburg | Neubrandenburg | Not required (non-interventional study) | N/A |
|  | Universitätsklinikum Münster | Münster | Not required (non-interventional study) | N/A |
|  | Nuc site of Universitätsklinikum Münster | Münster | Not required (non-interventional study) | N/A |
|  | Städtisches Klinikum Braunschweig | Braunschweig | Not required (non-interventional study) | N/A |
|  | Nuc site of Städtisches Klinikum Braunschweig | Braunschweig | Not required (non-interventional study) | N/A |
|  | Dr. Susan Feyerabend | Reutlingen | Not required (non-interventional study) | N/A |
|  | Universitätsklinikum Tübingen | Tübingen | Not required (non-interventional study) | N/A |
|  | Nuc site of Universitätsklinikum Tübingen (site 014) and | Tübingen | Not required (non-interventional study) | N/A |
|  | Praxis Dr. Feyerabend (site 013) | Erlangen | Not required (non-interventional study) | N/A |
|  | Nuc site of Universitätsklinikum Erlangen | Ulm | Not required (non-interventional study) | N/A |
|  | Nuc site of Universitätsklinikum Ulm | Magdeburg | Not required (non-interventional study) | N/A |
|  | Universitätsklinikum Freiburg | Bonn | Not required (non-interventional study) | N/A |
|  | Universitätsklinikum Bonn | Bonn | Not required (non-interventional study) | N/A |
|  | Nuc site of Universitätsklinikum Bonn | Dessau | Not required (non-interventional study) | N/A |
|  | Nuc site of Diakonissenkrankenhaus Dessau | Frankfurt am Main | Ethkkommission der Universitätsklinikum Frankfurt | 19-May-15 |
|  | Nuc site of Universitätsklinikum Frankfurt | Göttingen | Ethikkommission der Universitätsmedizin Göttingen | 02-Jan-15 |
|  | Universitätsmedizin Göttingen | Göttingen | Ethikkommission der Universitätsmedizin Göttingen | 02-Jan-15 |
|  | Nuc site of Universitätsmedizin Göttingen | Homburg (Saar) | Not required (non-interventional study) | N/A |
|  | Universitätsklinikum des Saarlandes | Homburg (Saar) | Not required (non-interventional study) | N/A |
|  | Nuc site of Universitätsklinikum des Saarlandes | Kiel | Not required (non-interventional study) | N/A |
|  | Universitätsklinikum Schleswig-Holstein, Campus Kiel | Kiel | Not required (non-interventional study) | N/A |
|  | Universitätsklinikum Düsseldorf | Düsseldorf | Ethikkommission der Med. Fakultät der HHU Düsseldorf | 09-Dec-15 |
|  | Donau-Isar-Klinikum Deggendorf | Deggendorf | Not required (non-interventional study) | N/A |
|  | Helios Klinik Berlin-Buch | Berlin | Not required (non-interventional study) | N/A |
| Greece | Alexandra General Hospital of Athens | Athens | Alexandra General Hospital of Athens | 23-Mar-15 |
|  | University Hospital of Patra | Patras | University Hospital of Patra | 28-Jan-15 |
|  | University Hospital of Heraklion | Heraklion | University Hospital of Heraklion | 18-Feb-15 |
| Israel | Rambam MC | Haifa | Not required (non-interventional study) | N/A |
|  | Sourasky MC | Tel Aviv | Not required (non-interventional study) | N/A |
|  | Asaf Harofeh MC | Zrifin | Approval granted by the study site | 25-Sep-18 |
|  | Soroka MC | Beer Sheva | Not required (non-interventional study) | N/A |
|  | Wolfson MC | Holon | Approval granted by the study site | 17-Sep-18 |
| Italy | Fondazione IRCCS - Istituto Nazionale dei Tumori | Milano | CE Istituto Nazionale dei Tumori | 16-Dec-14 |
|  | Azienda Ospedaliera Arcispedale Santa Maria Nuova - IRCCS | Reggio Emilia | Comitato etico provinciale di Reggio Emilia | 07-Oct-15 |
|  | Azienda Ospedaliera – PO Annunziata | Cosenza | CE Regionale - Sezione Area Nord | 17-May-16 |
|  | Ospedale Sacro Cuore – Don Calabria | Negrar (VR) | CESC delle Provincie di Verona e Rovigo | 23-Sep-15 |
|  | IRST Istituto Scientifico Romagnolo per lo Studio e la Cura dei Tumori | Meldola (FC) | Comitato Etico Area Vasta Romagna | 23-Sep-15 |
|  | Ospedale Santa Chiara | Trento | Comitato etico per le Sperimentazioni Cliniche dell’Azienda Provinciale per i Servizi Sanitari di Trento | 26-May-16 |
|  | IRCCS CROB | Rionero in Vulture (PZ) | Comitato Etico Unico Regionale per la Basilicata | 04-Feb-16 |
|  | Policlinico G. Martino | Messina | Comitato Etico per Messina | 21-Sep-15 |
|  | AOU Policlinico di Modena | Modena | Comitato Etico Provinciale di Modena | 26-Feb-16 |
|  | AOU di Ferrara - Arcispedale Sant'Anna | Ferrara | Comitato Etico della Provincia di Ferrara | 19-Nov-15 |
|  | AOU Pisana – Ospedale Santa Chiara | Pisa | Comitato Etico Area Vasta Nord Ovest | 05-Nov-15 |
|  | AOU S. Luigi Gonzaga | Orbassano (TO) | CE Interaziendale A.O.U. S. Luigi Gonzaga di Orbassano | 09-Sep-15 |
|  | IFO - Istituto Regina Elena | Roma | CE Sezione IRCCS IFO – Fondazione G.B. Bietti | 09-Feb-16 |
|  | AOU Maggiore della Carità | Novara | CE Interaziendale A.O.U. Maggiore della Carità di Novara | 26-Oct-15 |
|  | Ospedale Molinette | Torino | CE Interaziendale A.O. “Città della Salute e della Scienza” di Torino | 24-Feb-16 |
|  | Istituto Europeo di Oncologia | Milano | CE Istituto Europeo di Oncologia | 09-Sep-15 |
|  | A.O. Papa Giovanni XXIII | Bergamo | CE della Provincia di Bergamo | 22-Feb-16 |
|  | Centro di Riferimento Oncologico | Aviano (PN) | Comitato Etico Indipendente CRO | 29-Feb-16 |
|  | Ospedale Centrale di Bolzano | Bolzano | Comitato etico Bolzano | 22-Jun-16 |
|  | stituto Nazionale Tumori IRCCS "Fondazione G. Pascale" | Napoli | CE IRCCS Pascale | 16-Dec-15 |
|  | Ospedale San Carlo | Potenza | Comitato Etico Unico Regionale per la Basilicata | 26-Jan-16 |
|  | Ospedale Monaldi | Napoli | CE SUN e AO dei Colli | 28-Jan-16 |
|  | Istituti Ospitalieri | Cremona | CE dell'Area di Cremona, Lodi e Mantova | 05-May-16 |
|  | Spedali Civili | Brescia | CE Provinciale della provincia di Brescia | 19-Feb-16 |
|  | Ospedale Sant'Andrea | Roma | CE dell’Università “Sapienza” (Policlinico Universitario Umberto I - Azienda Ospedaliera S. Andrea) | 23-Jun-16 |
|  | AO Sant'Orsola Malpighi | Bologna | Comitato Etico per Bologna | 20-Apr-16 |
|  | Ospedale Civile Spirito Santo | Pescara | CE per la Ricerca Biomedica delle Province di Chieti e di Pescara e dell’Università degli Studi "G. D’Annunzio" di Chieti e Pescara | 11-Nov-16 |
| Luxembourg | Centre Hospitalier de Luxembourg | Luxembourg | Comité National d'éthique de Recherche (CNER) | 09-Jul-14 |
|  | Centre Hospitalier Du Nord, Clinique Saint-Louis | Ettelbrück | Not required (non-interventional study) | N/A |
|  | Centre Hospitalier Emile Mayrisch | Esch-sur-Alzette | Not required (non-interventional study) | N/A |
| Mexico | Torre 2 Consultorio 625-Hospital Medica Sur | Mexico | Comité de Medica Sur SAB de CV | 11-Apr-16 |
|  | Hospital Christus Muguerza Sur. Consultorio 707 | Monterrey Nuevo León | Grupo Christus Muguerza/Comité Medica Sur SAB de CV | 29-Feb-16 |
|  | Hospital Christus Muguerza. Hospital de Alta Especialidad | Monterrey Nuevo León | Grupo Christus Muguerza/Comité Medica Sur SAB de CV | 29-Feb-16 |
| Netherlands | Tweesteden ziekenhuis | Tilburg | Not required (non-interventional study) | N/A |
|  | Zaans Medisch Centrum | Zaandam | Not required (non-interventional study) | N/A |
|  | UMCG | Groningen | Not required (non-interventional study) | N/A |
|  | Albert Schweitzer Ziekenhuis | Dordrecht | Not required (non-interventional study) | N/A |
| Portugal | Fundação Champalimaud | Lisboa | Comissão de Ética Fundação Champalimaud | Apr-15 |
| Spain | HOSPITAL LA PAZ | Madrid | EC approval from other site (H. CLINICO SAN CARLOS) accepted | N/A |
|  | H. PUERTA DEL HIERRO | Majadahonda | EC H. PUERTA DEL HIERRO | 25-May-15 |
|  | H. DEXEUS QUIRON | BARCELONA | EC GRUPO HOSPITALARIO QUIRON | 08-Jun-15 |
|  | H. U. SANT JOAN DE REUS | REUS | EC H UNIVERISTARI SANT JOAN DE REUS | 28-May-15 |
|  | HOSPITAL NTRA. SRA. LA CANDELARIA | Santa Cruz de Tenerife | EC H. NTRA SRA LA CANDELARIA | 03-Jun-15 |
|  | H.U.G.C. Dr. NEGRÍN | Las Palmas de Gran Canar | EC H.U.G.C DR NEGRIN | 28-May-15 |
|  | H. MARQUES DE VALDECILLA | Santander | EC INVESTIGACION CLINICA DE CANTABRIA IDIVAL | 26-Jun-15 |
|  | H. G.U. de CIUDAD REAL | Ciudad Real | EC H.G.U. CIUDAD REAL | 30-Jun-15 |
|  | H. SAN PEDRO DE ALCANTARA | CACERES | EC INVESTIGACION CLINICA DE CACERES | 24-Jun-15 |
|  | H. U. INFANTA CRISTINA | CACERES | EC approval from other site (H. CLINICO SAN CARLOS) accepted | N/A |
|  | H.de LEÓN | León | EC INVESTIGACION CLINICA DE LEON | 26-May-15 |
|  | H. U. BURGOS | BURGOS | EC INVESTIGACION CLINICA DEL AREA DE SALUD BURGOS Y SORIA | 26-May-15 |
|  | H. DR. PESET | VALENCIA | EC approval from other site (H. CLINICO SAN CARLOS) accepted | N/A |
|  | H. GENERAL DE VALENCIA | VALENCIA | EC H GENERAL DE VALENCIA | 01-Jun-15 |
|  | H SON ESPASES | PALMA DE MALLORCA | EC approval from other site (H. CLINICO SAN CARLOS) accepted | N/A |
|  | ICO Girona | GIRONA | EC H. JOSEP TRUETA | 29-Apr-16 |
|  | H. PROVINCIAL DE CASTELLÓN | Castellón de la Plana | EC CONSORCIO HOSPITALARIO PROVINCIAL DE CASTELLON | 26-Apr-16 |
|  | H. VIRGEN DE LA ARRIXACA | El Palmar, Murcia | EC approval from other site (H. CLINICO SAN CARLOS) accepted | N/A |
|  | H. MORALES MESEGUER | Murcia | EC H MORALES MESEGUER | 27-Apr-16 |
| Sweden | Länssjukhuset Ryhov | Jönköping | Regionala Etikprövningsnämnden i Linköping | 28-Jan-15 |
|  | Centralsjukhuset i Karlstad | Karlstad | Not required (non-interventional study) | N/A |
| United Kingdom | The Royal Sussex County Hospital | Brighton | Central ethics committee | 22-Nov-18 |
|  | The Christie NHS Foundation Trust | Manchester | Central ethics committee | 22-Nov-18 |
|  | St James University Hospital | Leeds | Central ethics committee | 22-Nov-18 |
|  | Nottingham City Hospital | Nottingham | Central ethics committee | 22-Nov-18 |
|  | Royal Marsden Hospital | London | Central ethics committee | 22-Nov-18 |
|  | Clatterbridge Hospital | Bebington | Central ethics committee | 22-Nov-18 |
|  | Maidstone Hospital | Maidstone | Central ethics committee | 22-Nov-18 |
|  | Churchill Hospital | Oxford | Central ethics committee | 22-Nov-18 |
|  | Royal Liverpool University Hospital | Liverpool | Central ethics committee | 22-Nov-18 |
| United States | GU Research Network, LLC | Omaha, Nebraska | Sterling | 22-Aug-14 |
|  | Advanced Radiation Centers of New York | Lake Success, New York | Sterling | 22-Sep-14 |
|  | Alaska Urological Institute | Anchorage, Alaska | Sterling | 21-Aug-14 |
|  | Maryland Oncology Hematology, P.A. | Rockville, Maryland | Sterling | 02-Sep-14 |
|  | Oregon Urology Institute | Springfield, Oregon | Sterling | 29-Sep-14 |
|  | 21st Century Oncology - Plantation, FL | Plantation, Florida | Sterling | 10-Sep-14 |
|  | 21st Century Oncology - Fort Myers, FL | Fort Myers, Florida | Sterling | 16-Sep-14 |
|  | 21st Century Oncology - Sarasota, FL | Lakewood Ranch, Florida | Sterling | 10-Sep-14 |
|  | Carolina Regional Cancer Center (21st Century Oncology) | Myrtle Beach, South Carolina | Sterling | 10-Sep-14 |
|  | Michigan Institute of Urology | Troy, Michigan | Sterling | 18-Sep-14 |
|  | Urology Associates, P.C. | Nashville, Tennessee | Sterling | 16-Oct-14 |
|  | Clinical Research Solutions | Middleburg Heights, Ohio | Sterling | 26-Sep-14 |
|  | Tulane | New Orleans, Louisiana | Local | 20-Nov-14 |
|  | Urology of Virginia, PLLC | Virginia Beach, Virginia | Sterling | 04-Nov-14 |
|  | Jersey Shore Medical Center | Neptune, New Jersey | Local | 11-Nov-14 |
|  | Pacific Shores Medical Group | Long Beach, California | Sterling | 11-Nov-14 |
|  | Penrose Cancer Center | Colorado Springs, Colorado | Local | 07-Oct-14 |
|  | Dana Farber Cancer Center | Boston, Massachusetts | Local | 29-Dec-14 |
|  | Seton Medical Center | Daly City, California | Local | 27-Jan-15 |
|  | Billings Clinic | Billings, Montana | Sterling | 15-Jan-15 |
|  | Central DuPage Hospital | Warrenville, Illinois | Local | 13-Oct-14 |
|  | Lynn Cancer Center | Boca Raton, Florida | Sterling | 10-Mar-15 |
|  | Stephenson Cancer Center, University of Oklahoma | Oklahoma City, Oklahoma | Local | 16-Dec-14 |
|  | Swedish Health Services | Seattle, Washington | WIRB | 20-Nov-14 |
|  | UC Davis Cancer Center | Sacramento, California | Local | 06-Jan-15 |
|  | Ralph H. Johnson VAMC | Charleston, South Carolina | Local | 19-Nov-15 |
|  | Northshore University Health System | Evanston, Illinois | Local | 27-Oct-14 |
|  | Roswell Park Cancer Institute | Buffalo, New York | Local | 04-Nov-14 |
|  | St. Louis University Medical Center | Saint Louis, Missouri | Local | 21-Aug-15 |
|  | Wake Forest Baptist Medical Center | Winston-Salem, North Carolina | Local | 30-Oct-14 |
|  | W.G.(Bill) Hefner Veterans Administration Medical Center | Winston-Salem, North Carolina | Local | 01-Oct-15 |
|  | University of Hawaii | Honolulu, Hawaii | WIRB | 04-Mar-15 |
|  | North Shore Hematology Associates | East Setauket, New York | Sterling | 06-Jan-15 |
|  | Southwest Cancer | Lubbock, Texas | Local | 18-Feb-15 |
|  | Piedmont Hospital | Atlanta, Georgia | WIRB | 02-Dec-14 |
|  | John Hopkins | Baltimore, Maryland | Local | 02-Nov-15 |
|  | University of VA Medical | Charlottesville, Virginia | Local | 06-Oct-15 |
|  | UTHSCSA (The University of Texas Health Science Center at San Antonio) | San Antonio, Texas | Local | 10-Feb-15 |
|  | GU Research Network - Wichita Urology Group | Wichita, Kansas | Sterling | 16-Apr-15 |
|  | Radiology Group of Abington | Abington, Pennsylvania | Local | 01-May-15 |
|  | MD Anderson - Cooper Health System | Township, New Jersey | Local | 31-Jul-15 |
|  | St. Louis VAMC | St. Louis, Missouri | Local | 22-Jun-15 |
|  | GU Research Network - Houston Metro Urology | Houston, Texas | Sterling | 24-Aug-15 |
|  | Scott and White Memorial Hospital and Clinic | Temple, Texas | Local | 27-Jan-16 |
|  | Ashland-Bellefonte Cancer Center | Kentucky | WIRB | 22-Jan-16 |
|  | Dartmouth - Norris Cotton Cancer Center | Lebanon, NH | Local | 17-Dec-15 |
|  | Chesapeake Urology Research Associates | Towson, MD | Sterling | 30-Nov-15 |
|  | DeKalb Medical Physicians Group Radiation Oncology Specialists | Decatur, georgia | Sterling | 14-Oct-15 |
|  | Cancer Specialists of North Florida | Jacksonville, Fl | Sterling | 22-Jan-16 |
|  | University of Washington | Seattle, Washington | WIRB | 13-Mar-16 |

N/A, not applicable

**Results from an ongoing study to see if drops in levels of alkaline phosphatase or improvements in pain during treatment with radium can predict better survival in patients with advanced prostate cancer**

This plain language summary has been prepared from an article published in the British Journal of Cancer, titled: “*Alkaline phosphatase decline and pain response as predictors of overall survival benefit in patients treated with radium-223: a post hoc analysis of the REASSURE study*”

**Background**

When men are diagnosed with advanced prostate cancer, it means that their cancer has spread to lymph nodes, bones, and/or other parts of the body. When cancer spreads to the bones, men often have a reduced quality of life due to issues such as pain.

Radium-223 (also known as radium) is a systemic radiation therapy that targets prostate cancer which has spread to the bones. Radium gets taken up by regions of bone surrounding the cancer cells and produces radiation that can kill these nearby cells.

Previous studies have shown that radium improves the median survival (the length of time from the start of treatment to when half of the patients were still alive) for men with advanced prostate cancer. Radium also improves quality of life and is well tolerated.

**Why was this research done?**

To decide whether a patient should continue radium, it is important to assess how their cancer responded to the treatment. This research looks at two possible factors for assessing responses to radium: pain and a protein called alkaline phosphatase (ALP).

- Pain is common in patients with advanced prostate cancer and can indicate worsening of the disease.
- ALP levels are sometimes monitored by doctors, as high levels may suggest that the cancer has spread to the bone.

If pain or ALP levels go down during treatment, it may suggest that the treatment is killing cancer cells in the bone.

Here we looked at whether it is possible to predict survival in patients receiving radium based on early changes in pain and ALP using data collected from REASSURE (an ongoing, worldwide study of radium in patients with advanced prostate cancer when prescribed by doctors as part of usual clinical practice).

**How was the research done?**

Patients were given radium as part of their normal treatment. Both ALP levels and pain were measured before treatment (also known as ‘baseline’). ALP levels were measured again after 12 weeks of treatment and pain was measured throughout radium treatment, to see if there were any changes. Patients were asked to rate their worst pain in the last 24 hours using a questionnaire. Pain was rated on a scale of 0 (no pain) to 10 (pain as bad as you can imagine). Patients who had a score of 2 or more at baseline were considered to be in pain. If a patient had a decrease in score of 2 or more during treatment, they were considered to have had a reduction in pain (i.e., a pain response).

**What were the results?**


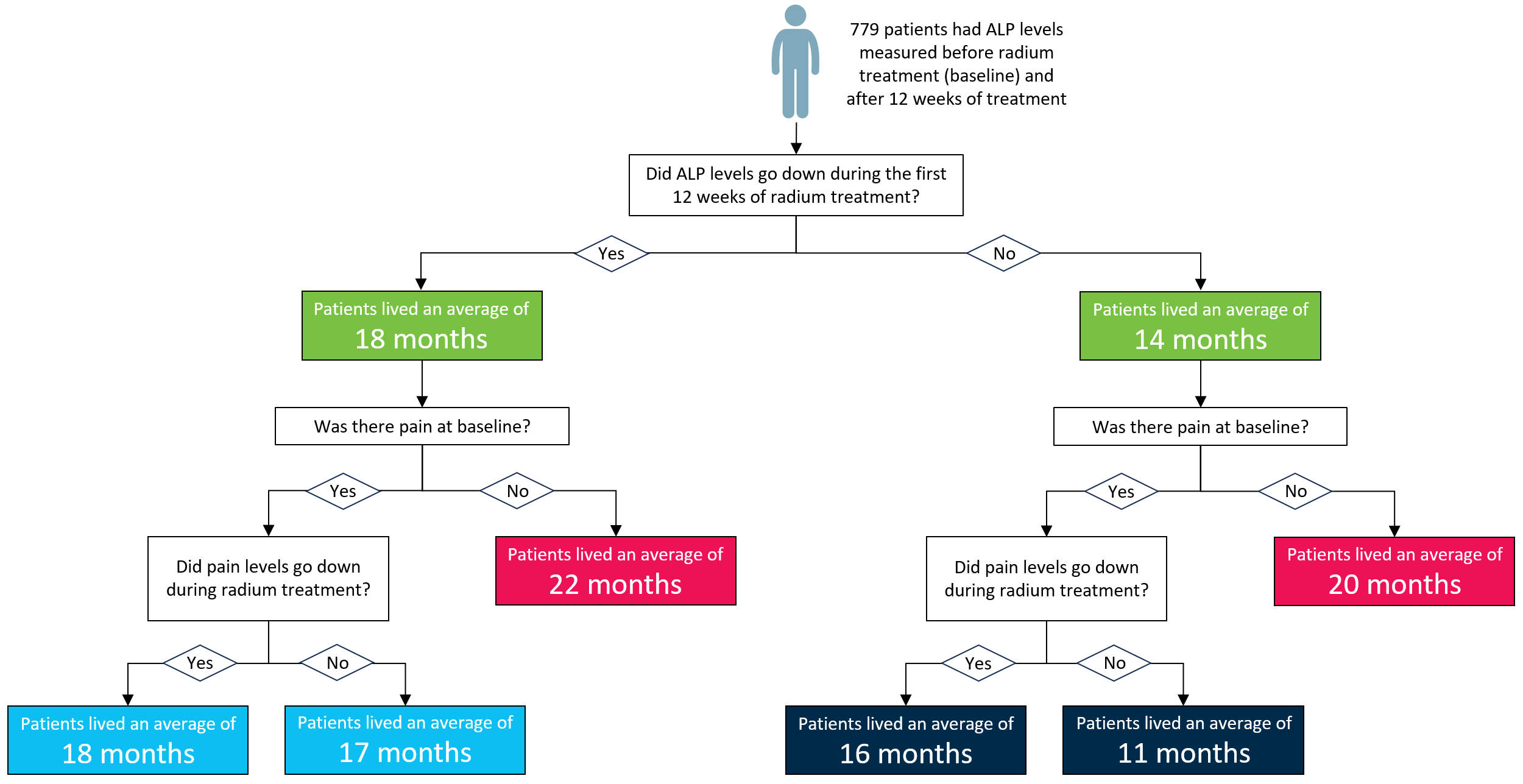


**What do the results show?**

ALP levels went down in eight out of ten (80%) patients during radium treatment.

- **Patients who had a drop in ALP levels during the first 12 weeks of radium treatment lived longer on average than those who did not (18 months versus 14 months, respectively).**

Around two out of ten (23%) patients had no pain at baseline.

- **For patients with no pain at baseline, survival was similar in those with or without a drop in ALP during the first 12 weeks of radium treatment (22 months versus 20 months, respectively).**

Around eight out of ten patients (77%) had pain at baseline.

- **Patients who had pain at baseline and a drop in ALP during the first 12 weeks of radium treatment lived for a similar length of time regardless of whether they had a reduction in pain during treatment or not (18 months versus 17 months, respectively).**
- **Patients who had pain at baseline but did not have a reduction in ALP during the first 12 weeks of radium treatment lived longer if their pain reduced during treatment than if it did not (16 months versus 11 months, respectively).**

**What does this mean?**

These results suggest that, when making decisions with their patients, it may be helpful for doctors to consider how ALP and pain levels change during treatment with radium.

For more information on the study (Trial registration: ClinicalTrials.gov identifier NCT02141438), click [here](https://clinicaltrials.gov/study/NCT02141438?term=NCT02141438&rank=1)

**Acknowledgements**

The authors would like to thank the patients, investigators, and study teams involved in this study.

**Disclosures and disclaimers**

This study was sponsored by Bayer AG. Radium is approved to treat the condition discussed in this summary. This summary is intended for informational use only and is not intended to promote any product. Writing support for this summary was provided by Chris Guise of Cancer Communications and Consultancy Ltd, Cheshire, UK (funded by Bayer AG).
